# Neuronally-directed effects of RXR activation in a mouse model of Alzheimer’s disease

**DOI:** 10.1038/srep42270

**Published:** 2017-02-16

**Authors:** M. M. Mariani, T. Malm, R. Lamb, T. R. Jay, L. Neilson, B. Casali, L. Medarametla, G. E. Landreth

**Affiliations:** 1 Alzheimer Research Laboratory, Department of Neurosciences, School of Medicine, Case Western Reserve University, Cleveland, OH 44106, USA; 2 A.I. Virtanen Institute for Molecular Sciences, Department of Neurobiology, University of Eastern Finland, Neulaniementie 2, 70211 Kuopio, Finland

## Abstract

Alzheimer’s disease (AD) is characterized by extensive neuron loss that accompanies profound impairments in memory and cognition. We examined the neuronally directed effects of the retinoid X receptor agonist bexarotene in an aggressive model of AD. We report that a two week treatment of 3.5 month old 5XFAD mice with bexarotene resulted in the clearance of intraneuronal amyloid deposits. Importantly, neuronal loss was attenuated by 44% in the subiculum in mice 4 months of age and 18% in layer V of the cortex in mice 8 months of age. Moreover, bexarotene treatment improved remote memory stabilization in fear conditioned mice and improved olfactory cross habituation. These improvements in neuron viability and function were correlated with significant increases in the levels of post-synaptic marker PSD95 and the pre-synaptic marker synaptophysin. Moreover, bexarotene pretreatment improved neuron survival in primary 5XFAD neurons *in vitro* in response to glutamate-induced excitotoxicity. The salutary effects of bexarotene were accompanied by reduced plaque burden, decreased astrogliosis, and suppression of inflammatory gene expression. Collectively, these data provide evidence that bexarotene treatment reduced neuron loss, elevated levels of markers of synaptic integrity that was linked to improved cognition and in an aggressive model of AD.

Alzheimer’s disease (AD) is a highly prevalent disorder characterized by progressive cognitive impairment associated with the accumulation of amyloid beta (Aβ) within the brain and subsequent development of neuronal dystrophy and death. In experimental models of AD, treatment with nuclear receptor agonists results in improved cognition and memory and attenuation of the disease-related pathology^1^. Nuclear receptors are ligand activated transcription factors which directly bind to enhancer and promoter elements within their target genes which act broadly to regulate cellular energy and lipid metabolism and to suppress tissue inflammation^2^,^3^,^4^. In the brain, the principal type II nuclear receptors are peroxisome proliferator activated receptors gamma and delta (PPARγ, PPARδ) and Liver X Receptors (LXRs)^1^. PPARs and LXRs form obligate heterodimers with retinoid X receptors (RXRs), forming a functional transcription factor. The transcriptional activity of these dimeric receptors can be stimulated by ligation of either member of the receptor pair. In murine models of AD and neuroinflammation, studies of the effects of nuclear receptor agonists have been focused principally on their actions in astrocytes and microglia^5^,^6^,^7^,^8^. However, it has recently been appreciated that these nuclear receptors exhibit a broad range of neuronally-directed actions^9^,^10^,^11^,^12^,^13^,^14^. Our primary objective was to ascertain if nuclear receptor activation would attenuate the neuronal dysfunction and loss in a murine model of AD. The most commonly used murine models of AD do not exhibit disease-related neuronal loss^15^. We have employed 5XFAD mice which express five familial Alzheimer’s disease (FAD) mutations in APP and PS1 under the neuron specific mouse Thy-1 promoter^16^. The 5XFAD transgenic mice exhibit intraneuronal deposits of amyloid precursor protein (APP), and its processing products, including Aβ peptides (hereafter termed APP/Aβ). These intraneuronal accumulations of APP/Aβ appear in neurons in layer V and the subiculum of 5XFAD mice early in disease pathogenesis before extracellular plaques form^16^,^17^. Moreover, these mice have robust neuritic dystrophy, extracellular amyloid deposition, and gliosis. Importantly, the model exhibits neuronal death in pyramidal neurons in the subiculum and layer V of the cortex, at 4 and 8 months of age, respectively as well as behavioral deficits^16^,^17^. It has been postulated that extracellular plaques in 5XFAD mice arise from neurons that have undergone apoptosis due to internally accumulated APP/Aβ and serve as the nidus for plaque formation^17^,^18^. However, the ultimate cause of neuronal demise is unknown.

We have employed an RXR agonist, bexarotene, which acts to regulate gene expression in the brain^9^,^12^. Bexarotene has been reported to improve memory, cognition, and pathology in mouse models of AD^8^,^19^ and aging^11^ and is now in early phase clinical trials^20^,^21^. Moreover, bexarotene has recently been shown to be effective in murine models of Parkinson’s^22^, ALS^23^, multiple sclerosis^24^ and stroke^25^.

In the present study, we demonstrate that bexarotene treatment increases neuron survival in the 5XFAD mice. Furthermore, we demonstrated bexarotene-induced intraneuronal reduction with concomitant behavioral improvements in olfactory cross habituation and remote memory stabilization. Lastly, we have recapitulated bexarotene-dependent plaque removal in the 5XFAD mice.

## Results

### Intraneuronal APP and its processing products accumulate in Layer V neurons and are reduced upon bexarotene treatment

**Figure 1:**
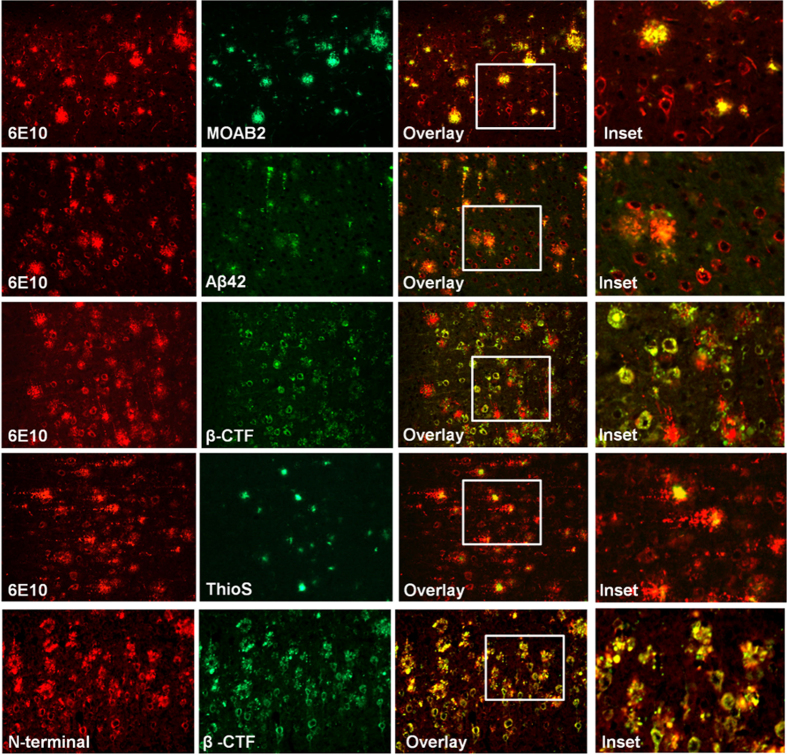
Intraneuronal deposits are primarily composed of APP and β-CTF. Representative image layer V cortical neurons from 4 month 5XFAD mice stained with 6E10, N-terminal APP antibody, β-CTF antibody, MOAB2, Aβ42, Aβ40, ThioS.

**Figure 2:**
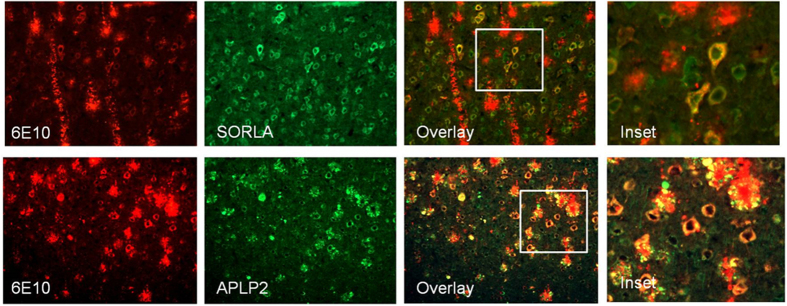
APP related proteins SORLA and APLP2 accumulate in 5XFAD neurons. Representative image layer V cortical neurons from 4 month 5XFAD stained with 6E10, SORLA, and APLP2.

The extensive deposition of Aβ within neurons has been reported in 5XFAD mice by several investigators^16^,^17^,^18^. The intraneuronal deposits are one of the most prominent features of this model, especially in the subiculum at young ages. Several studies have strongly linked accumulation of intraneuronal Aβ to neuron death^17^,^26^,^27^,^28^. However, the composition of intraneuronal Aβ-containing deposits in 5XFAD mice had not been firmly established. Therefore, we have examined the nature of the deposited species in order to obtain insight into the nature of the dysregulation of APP metabolism in the affected neurons. We characterized the intraneuronal amyloid species in 4 month old 5XFAD by using a battery of amyloid binding antibodies (N-terminal antibody, C-terminal antibody, MOAB, an antibody detecting oligomeric Aβ, Aβ40, and Aβ42) to identify the main species present within neurons in the cortex. The neuronal soma exhibited extensive immunoreactivity to 6E10, which recognizes the Aβ1-16 epitope present in full length APP and Aβ peptides. We found immunoreactivity to both N-terminal and β-CTF specific antibodies in the cells located in the cortex (Fig. 1). However, examination of Aβ42 deposition, the principal Aβ peptide present in this model, revealed that this species represented only a small fraction of the material detected intraneuronally and was found with a punctate distribution, as reported previously (Fig. 1)^13^. This latter observation corroborates that of Eimer *et al*.^18^. The robust 6E10 immunoreactivity suggests intraneuronal APP/Aβ processing products are accumulating within neurons, reflecting a generalized impairment of protein processing and trafficking in the affected neurons. To address this point, we examined APLP2, a protein related to APP but not overexpressed in this mouse model. APLP2 was accumulated in the same neurons bearing elevated levels of 6E10 immunoreactivity and with a similar distribution (Fig. 2). Additionally, we evaluated levels of SORLA within 6E10 positive neurons. SORLA belongs to the vacuolar protein sorting 10 (VPS10) domain receptor family and functions as an intracellular sorting and trafficking receptor for APP. Typically, SORLA is found on endosomes, however we observed the extensive accumulation of this protein throughout the 6E10 positive neurons in the cortex (Fig. 2), reflecting the extensive dysregulation of neuronal vesicular trafficking. We did not observe transgene or treatment dependent effects on the expression of Rab 5 or Rab 7 (Supplemental Fig. 1).

**Figure 3:**
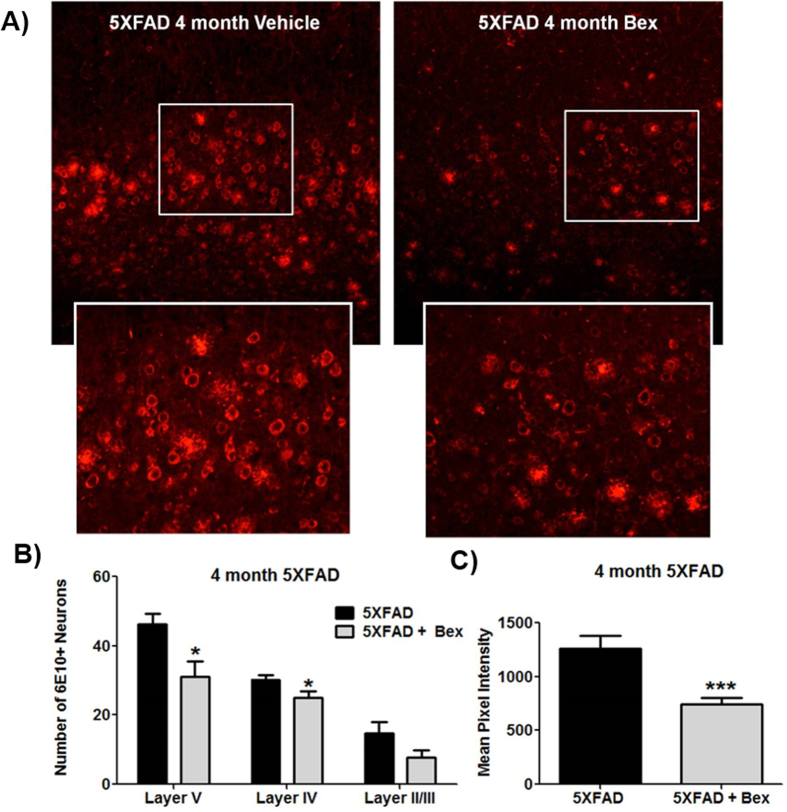
Intraneuronal APP/Aβ is reduced after bexarotene treatment. (**A**) Representative image layer V cortical neurons from 4 month 5XFAD after 15 days of vehicle or bexarotene treatment stained with 6E10. (**B**) Blinded counts were performed on 6E10 stained cortices (**C**) and pixel intensity was measure by Image J. Student’s T test, *p < 0.05 ***p < 0.001.

A blinded analysis was conducted to assess the mean fluorescence intensity of individual neuron’s 6E10 staining, as well as the number of 6E10 positive neurons. We examined 4 month old 5XFAD mice since the abundance of plaques at 8 months obscures intraneuronal APP/Aβ, as seen in other mouse models with intraneuronal APP/Aβ^29^,^30^. Importantly, we observed that intraneuronal 6E10 staining was diminished in the bexarotene treated mice (Fig. 3). We found that both the number of 6E10 positive neurons in layer V and their mean fluorescence intensity were reduced after bexarotene treatment (Fig. 3). There was a quantitatively smaller, but significant reduction in Layer IV neurons, and no effect was observed in layer II/III, consistent with the sparing of the upper layer neurons reported by Oakley *et al*.^16^.

We did not consistently observe bexarotene-induced changes in markers of autophagy (LC3-II and p62/SQSTM1; Supplemental Fig. 1).

### Improved neuron survival in subiculum and layer V cortex after bexarotene treatment

**Figure 4:**
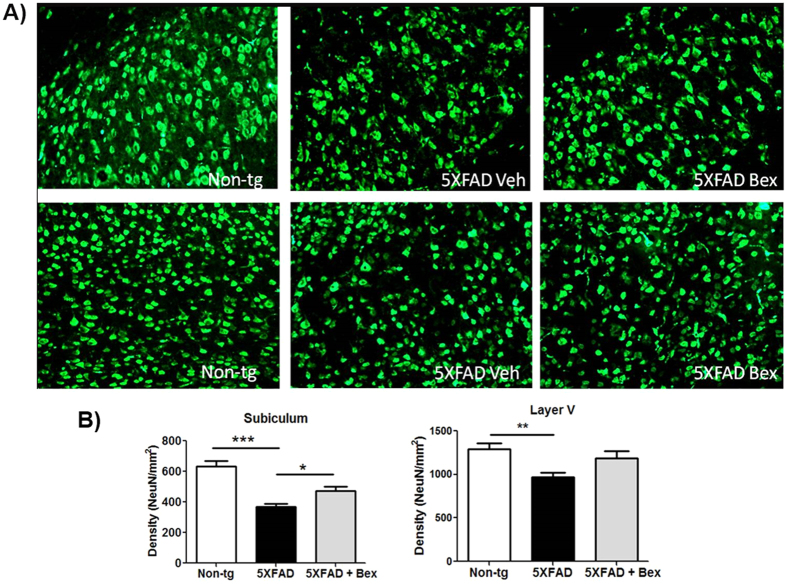
Bexarotene improves neuron survival in subiculum and layer V cortex. (**A**) Representative images from 4 month subiculum or 8 month layer V cortex of non-transgenic or 5XFAD mice with bexarotene or vehicle after 15 days of treatment stained with NeuN. (**B**) NeuN+ cells density was determined in the subiculum of 4 month 15 day treated 5XFAD mice or layer V of the cortex in 8 month 15 day treated 5XFAD mice, One-way ANOVA, *p < 0.05.

We assessed whether bexarotene treatment would attenuate neuronal death in the subiculum and cortical lamina V in 5XFAD mice. In the subiculum there is an approximate 40% loss of neurons over the period from 2–4 months of age^17^. Whereas, in the cortex, there is a selective loss of neurons in cortical lamina V that occurs between 8–12 months of age^16^,^18^. 5XFAD mice were treated with bexarotene for 15 days beginning at either 3.5 or 7.5 months of age and the number of neurons was determined my manually counting neurons in either the subiculum or layer V of the cortex, respectively. 5XFAD mice exhibited 39% fewer neurons in the subiculum compared to non-transgenic mice (Fig. 4), consistent with previous reports^17^. However, the number of neurons in the subiculum of bexarotene treated 5XFAD mice was only reduced by 22% (Fig. 4). Thus bexarotene treatment increased survival of subicular neurons by 44% with two weeks of drug treatment. Examination of neuronal number in layer V of the cortex at 8 months of age, revealed a loss of 27% of these neurons in the 5XFAD mice compared to non-transgenic controls. Bexarotene treatment modestly attenuated the neuronal loss (18%), but this trend did not reach significance (Fig. 4). The reduced effect size in the cortex is likely reflective of the longer period during which neuronal loss occurs relative to the short period of drug treatment.

**Figure 5:**
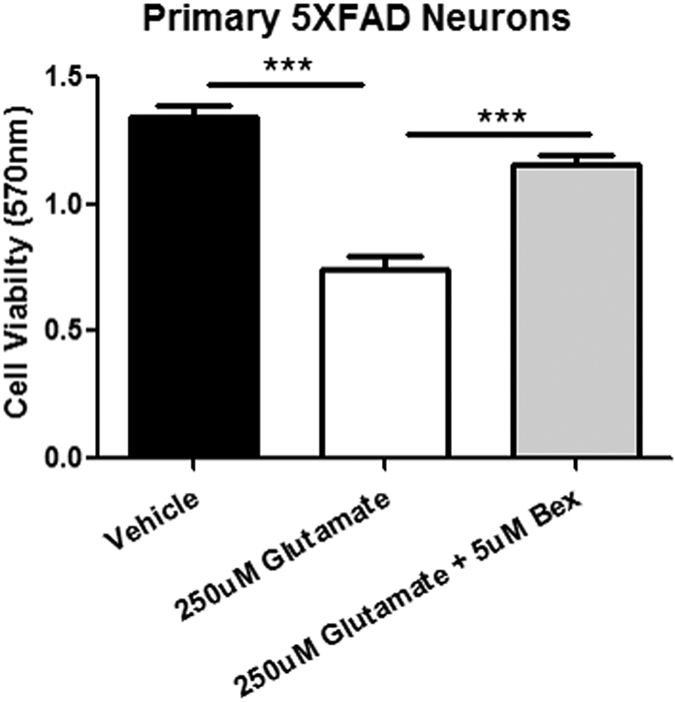
Bexarotene pretreatment improves primary 5XFAD neuron survival during glutamate excitotoxicity. Primary 5XFAD neurons were treated with neurobasal media alone or 5 uM bexarotene for 24 hours. Then media was replaced with neurobasal media alone, 250 uM glutamate, or 250 uM glutamate with 5 uM bexarotene. After 24 hours, supernatant was removed and MTT viability assay was performed. ANOVA with Tukey post-hoc test; ***p < 0.001.

We extended these studies to ascertain if analogous effects were observed *in vitro*, as it remained a possibility that bexarotene might exert its effects on neuronal survival indirectly. Cortical neuronal cultures from 5XFAD mice were subject to glutamate excitotoxicity. Glutamate treatment resulted in loss of approximately 50% of the neurons (Fig. 5). However, if the neurons were exposed to bexarotene for 24 hrs prior glutamate treatment the neuronal loss was dramatically attenuated (Fig. 5). These data provide evidence for a direct neuroprotective effect of bexarotene and independent of any glial influences.

### Bexarotene improved remote memory stabilization and olfactory cross habituation

**Figure 6:**
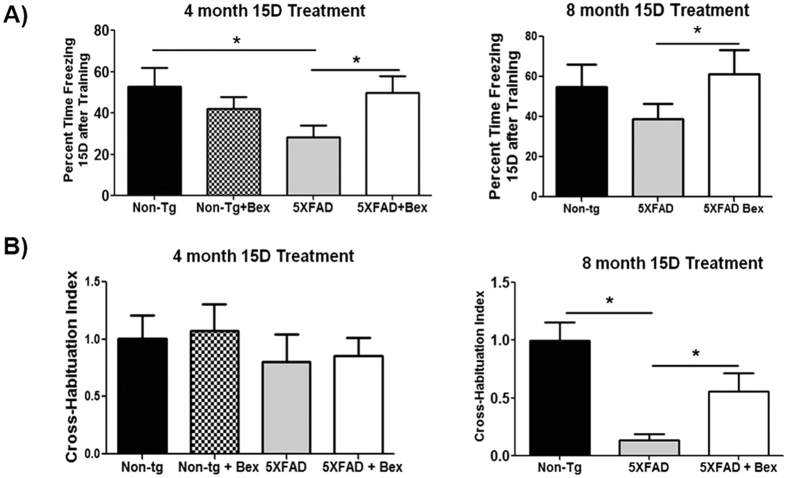
Remote memory stabilization and olfaction cross habituation are improved after bexarotene treatment. 4 and 8 month 5XFAD mice and littermate control mice were treated with vehicle or bexarotene. (**A**) Fourteen days after training, mice were tested for freezing response for five minutes. (**B**) Four unique odors were presented to 5XFAD mice in 4 successive trials. Cross-habituation is seconds spent sniffing from Trial 1 of a novel odor subtracted by sniffing the previous odor in Trial 4. Student’s T test, *p < 0.05 (n = 8–15).

We tested if the bexarotene-induced neuron survival correlated with improved cognition during remote memory stabilization and olfactory cross habituation. Since previous reports have found that 5XFAD mice do not have short term reconsolidation deficits, we quantified remote memory stabilization over 14 days using a fear conditioning assay optimized by Kimura *et al*. for the 5XFD model^31^. During training, mice were placed in a conditioning chamber for 12 min and received four footshocks (1.0 mA, 2 s). Remote memory stabilization was evaluated by scoring freezing behavior for 5 min when the mice were placed back into the same conditioning chamber 14 days after training to measure hippocampus-dependent fear memory formation. 5XFAD mice have significantly reduced freezing time 14 days after training at either 4 or 8 months of age which has also been reported in 30 day remote memory stabilization indicating deficits in long term recall^31^ (Fig. 6). This deficit was significantly improved in the bexarotene treated group at either 4 or 8 months of age (Fig. 6).

We examined the effect of bexarotene in a short-term behavioral test, olfactory cross-habituation, a measure of odor discrimination. Deficiencies in olfactory cross-habituation indicate incorrect decoding in the piriform cortex from sensory input from the olfactory bulb due to Aβ^32^. Additionally, anosmia can be an early sign of pathology in Alzheimer’s disease^33^,^34^. Olfaction cross-habituation deficits were first detected first at 8 months of age, since 4 month 5XFAD mice did not display any olfaction cross-habituation deficits (Fig. 6). 5XFAD mice at 8 months of age had significant impairments in distinguishing odors (discrimination) which was significantly improved after bexarotene treatment (Fig. 6).

### 5XFAD mice have increased levels of pre- and post-synaptic markers after bexarotene treatment

**Figure 7:**
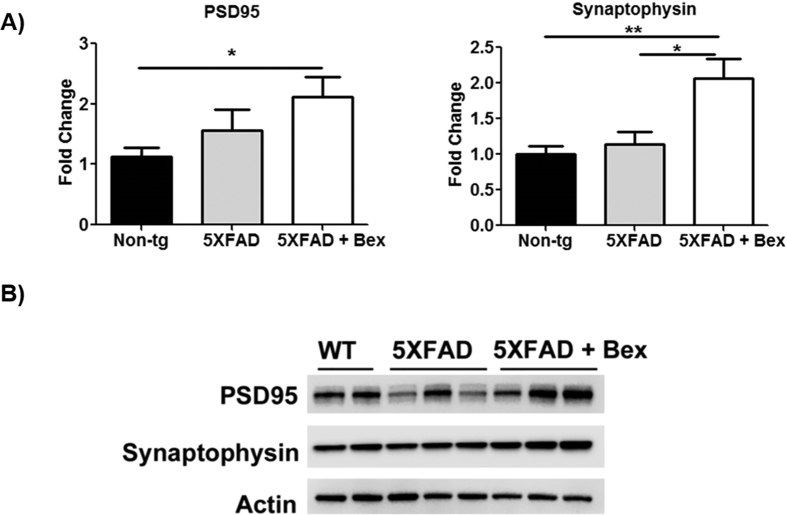
Pre- and post- synaptic markers are increased after bexarotene treatment. (**A**) 4 month 5XFAD hippocampal and cortical homogenates were analyzed for the expression of PSD95 and synaptophysin by Western analysis and quantified with Image J. One-way ANOVA, *p < 0.05 (**B**) Representative Western blot for PSD95, synaptophysin, and actin.

We explored whether the improved behavioral responses were reflected in abundance of synaptic proteins that are reduced in this model^16^. Investigation of the underlying mechanism of bexarotene treatment has recently begun to address its role in neurogenesis and synaptic plasticity^11^,^12^. Therefore, we examined PSD95 and synaptophysin in 15 day bexarotene treated mice. At 4 months of age the 5XFAD do not have a PSD95 or synaptophysin deficit (Fig. 7). However, bexarotene treatment increased PSD95 and synaptophysin protein levels (Fig. 7). These findings are consistent with those of Tachibana *et al*. who found that 8 weeks of bexarotene treatment in 20–24 month aged non-transgenic mice significantly increased both PSD95 and synaptophysin^11^. Moreover, Mounier *et al*. demonstrated bexarotene enhanced dendritic complexity by increased branching and intersections in primary neurons^12^.

### Bexarotene reduced inflammation and astrogliosis

**Figure 8:**
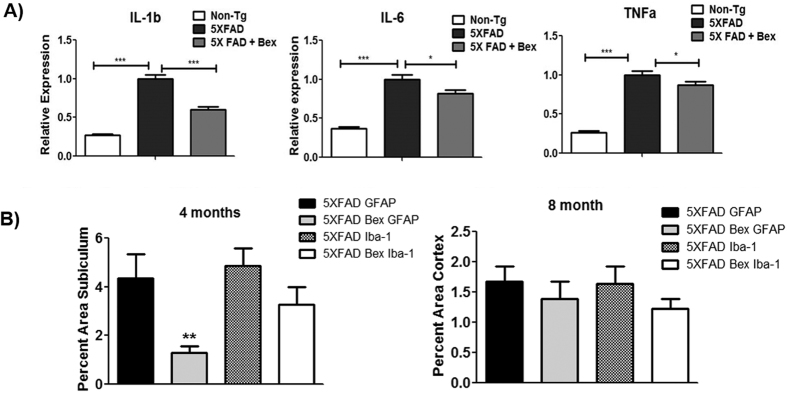
Bexarotene reduced inflammatory cytokines and astrogliosis. (**A**) Quantification of mRNA levels in combined cortex and hippocampal homogenates of 4 month 5XFAD mice treated for 15 days with bexarotene. All statistics were done at the ΔCt level using the ΔCt ± S.E.M. (**B**) GFAP and Iba-1 area was quantified with ImagePro Premier in the subiculum of the 4 month 5XFAD mice and cortex of the 8 month 5XFAD mice with vehicle or bexatorene treatment. Samples were compared using one-way ANOVA with Tukey post-hoc test; n = 8–10 animals. *p < 0.05, **p < 0.01, ***p < 0.001.

To form a complete picture of the neuronal environment, we examined gliosis and inflammation in the 4 month old 5XFAD mice. Nuclear receptor agonists have been demonstrated to exhibit potent anti-inflammatory agents in murine models of AD^1^. We examined classical mediators of inflammation IL-6, IL-1β, and TNF-α through rt-PCR analysis of mRNA levels in cortical and hippocampal brain homogenates. After 15 days of treatment, bexarotene decreased all three inflammatory mediators examined (Fig. 8). As the producers of these inflammatory mediators in the brain, microglia and astrocytes were examined. Iba-1 area, representative of microglia and brain infiltrating macrophages, was not significantly reduced. In contrast, GFAP area was significantly reduced in the subiculum with drug treatment (Fig. 8).

### Bexarotene treatment reduces amyloid plaque accumulation but not Aβ42 in 5XFAD mice

Bexarotene has previously been shown to reduce amyloid plaque pathology in APP/PS1, APPPS1–21, and Tg2576 mouse models of AD^8^. This effect has recently been shown to result from the actions of nuclear receptor agonists on plaque-associated macrophages, which licensed and stimulated their phagocytic actions^6^. However, it was unknown if bexarotene would be effective in a very aggressive model of model of amyloidosis with neuronal death. 5XFAD mice aged 3.5 and 7.5 months were treated with bexarotene for 15 days and plaque pathology was analyzed through 6E10 staining. Plaque area was significantly reduced upon treatment in either the subiculum at 4 months of age or in the cortex at 8 months of age (Supplemental Fig. 2). As expected, bexarotene treatment increased APOE expression and lipidation status, as well as, ABCA1 and ABCG1 expression providing a positive control for drug action in the brain (Supplemental Fig. 2, data not shown)^8^.

The antibody 6E10 binds full length APP, Aβ40, and Aβ42, and therefore, cannot be used to distinguish amyloid species. ELISA was used to determine if bexarotene altered soluble and insoluble Aβ 40/42 levels in combined cortical and hippocampal homogenates. We report no significant reductions in Aβ40 and Aβ42 in our 15 day treatment paradigm in 4 month 5XFAD (Supplemental Fig. 3). Interestingly, at 8 months of age, 15 day treatment significantly reduced both soluble and insoluble Aβ40 (Supplemental Fig. 3).

## Discussion

Here we report that bexarotene improves neuron survival and reduces intraneuronal APP/Aβ deposition in an aggressive mouse model of Alzheimer’s disease. Additionally, we have characterized the composition of the intraneuronal deposits using a comprehensive array of antibodies and determined that the prevalent species are full length APP and β-CTFs, with Aβ42 comprising a minor constituent of the intraneuronal accumulations.

There is mounting evidence that endosomal, lysosomal, and autophagic processes are functioning aberrantly in the 5XFAD mice. 5XFAD mice have reduced cathepsin D activity and decreased N-glycosylation of v-ATPase, which is indicative of elevated lysosomal pH and impairment of normal proteolytic degradation of proteins including Aβ^35^. Treatment with a GSK-3 inhibitor restored lysosomal acidification and enhanced Aβ clearance in 5XFAD brains, although its effects on intraneuronal APP/Aβ were not examined^35^. There is evidence that PS1 mutations are responsible for the lack of acidity in lysosomes in mouse models of AD, impairing protein degradation. The Nixon laboratory has demonstrated that PS1 is essential for v-ATPase targeting to lysosomes and lysosome acidification^36^,^37^. The lack of lysosomal acidification may contribute to the many reported accumulations of APP/Aβ. Ohno and colleagues identified full-length APP and β-CTF in the mitochondria of 5XFAD mice. Even with partial deletion of BACE1 considerable amounts of APP and β-CTF accumulated in mitochondria of 12-month-old 5XFAD mouse brains^38^. Moreover, Eimer *et al*. characterized accumulation of intraneuronal Aβ42 throughout the endosomal-lysosomal system with LAMP-1 and the transferrin receptor co-localized with Aβ42^18^. Autophagy may also be increased or improperly functioning, as evidenced by detection of elevated LC3B-II protein levels^39^. However, we were unable to detect consistent changes in other autophagic proteins, thus ability of bexarotene to broadly stimulate autophagy remains speculative.

We demonstrated accumulation of non-FAD proteins, SORLA and APLP2, in 5XFAD neurons. Neither SORLA nor APLP2 is overexpressed in the 5XFAD mouse, yet pyramidal neurons display florid co-staining with 6E10 (Fig. 1). SORLA is an endocytic receptor that binds to APP and shuttles it between the Golgi apparatus, the plasma membrane, and endosomes. Additionally, SORLA has recently been shown to deliver Aβ to lysosomes^40^. APLP2, amyloid precursor-like protein 2, is highly homologous to APP and is expressed in neurons. APLP2, like APP, exhibits robust accumulation with the same neuronal vesicular compartments, reflecting a generalized dysregulation of the endolytic system that is ameliorated by bexarotene treatment. It is possible that bexarotene is exerting beneficial effects on vesicular trafficking of endosomes. Lee *et al*. demonstrated that apoE increased endosomal trafficking to lysosomes in microglia, and is perhaps having a similar effect in neurons^30^.

ApoE, a critical cholesterol transport protein, has been implicated as a vital factor for synaptic plasticity, neuronal activity, and injury repair^12^,^41^,^42^. Our work and that of others had previously focused on the roles of apoE in mediating the clearance of soluble forms of amyloid from the interstitial fluid, and in this way ameliorated the effects of Aβ peptides on synaptic activity and network function^8^,^10^. The recent study by Tachibana *et al*. provides critical new insight into the roles of apoE in neuron viability. As a primary target of bexarotene, apoE may contribute to improved synaptic health through increased signaling through LRP1. Tachibana *et al*. have clearly demonstrated that bexarotene increased pre- and post-synaptic marker expression in aged mice but not in bexarotene treated nLrp1−/− mice^11^. Importantly, apoE binds to a variety of other neuron and glial surface receptors including LDLR, VLDLR, and ApoER2, and could be exerting beneficial signaling through these receptors^43^. Further evidence of the importance of apoE in synaptic plasticity and neurogenesis has been obtained by characterizing neurons expressing the apoE4 isoform. Mice expressing the human apoE4 isoform have synaptic deficits and impairment in long-term potentiation, memory and cognition^41^. Boehem-Cagan *et al*. reported enhanced apoE lipidation in E4-knock-in mice, restored cognitive performance, and increased synaptic markers with bexarotene treatment^9^. Moreover, bexarotene treatment decreased accumulation of Aβ42 and hyperphosphorylated tau in hippocampal neurons, and increased vesicular glutamatergic transporter 1 (VGluT1) in the *APOE4*-knock-in mice^9^. Mounier *et al*. found that bexarotene enhanced dendritic complexity in primary neurons, and improved dendritic structure in the hippocampus of human *APOE4* knock in mice which had deficits in dendritic branching^12^. Collectively, these data indicate that apoE is critical for neuron structure and function, and that increased production or lipidation of apoE through bexarotene administration can overcome apoeE4-induced deficits.

Due to the pleotropic nature of bexarotene action, there are likely multiple mechanisms contributing to reductions in intraneuronal APP/Aβ and enhanced neuronal survival. PPARγ has also been reported to reduce intraneuronal amyloid in APPV7171 and 3XTg mouse models of AD following Pioglitazone treatment^44^,^45^. In contrast, we have recently reported that PPARδ agonist GW0742 did not reduce intraneuronal 6E10 immunoreactivity in layer V neurons^7^. These data suggest that bexarotene may act to facilitate intraneuronal APP/Aβ removal through activation of RXR:PPARγ. Additionally, Savage *et al*. demonstrated bexarotene treatment improved *ex vivo* slice phagocytosis of microglia and macrophage in APP/PS1Δe9 mice, and reduced plaque burden in the hippocampus *in vivo*^6^. Thus, indicating that bexarotene-dependent plaque reduction may be due to increased phagocytois by microglia and macrophages. The microglia and macrophage-mediated reduction in plaques near neurons may have contributed to the reduced inflammatory gene expression in the neuron environment (Fig. 8). Furthermore, the reduction in GFAP positive astrocytes and accompanying inflammatory cytokine production could contribute to enhanced neuron survival (Fig. 8). However, there is no proposed glial mechanism that would cause reductions in intraneuronal 6E10^+^ deposits.

We report that bexarotene treatment of 5XFAD mice resulted in a reduction in plaque burden (Supplemental Fig. 2), replicating findings reported in Cramer *et al*. in other murine models of the disease^8^. Subsequent studies reproduced the ability of bexarotene to reverse AD-related behavioral deficits^9^,^19^,^46^,^47^. However, other laboratories were unable to replicate the loss of plaques and the reduction of soluble forms of Aβ was variably observed, including studies in the 5XFAD mice^48^. A review of this literature by Tesseur and DeStrooper documented that a critical difference between these studies was the drug formulation^46^ which dramatically affect the pharmacodynamics of bexarotene^49^. The FDA approved formulation (Targretin TM) is a microcrystalline form which is slowly absorbed, compared to solubilized preparations which are rapidly cleared. We have subsequently dissected the mechanism through which bexarotene stimulated the phagocytic clearance of Aβ plaques^6^.

Bexarotene has recently been reported to have salutary effects on a number of animal models of CNS disorders including models of AD, ALS, Parkinson’s disease, multiple sclerosis epilepsy, hypertension, and stroke^9^,^10^,^22^,^23^,^50^,^51^. Significantly, McFarland reported that bexarotene acted to stimulate the activity of the nuclear receptor Nurr1 in an animal model of PD through Nurr1-RXR heterodimers^22^. These authors treated 6-hydroxydopamine (6-OHDA) lesioned rats with very low dose bexarotene which resulted in increased retention of dopamine neurons and improved behavioral performance^22^. Bomben *et al*. demonstrated that bexarotene supressed Aβ-dependent increases in control network hyperexcitability and reduced seizures in Kv1.1 mice^10^. These data corroborate our own data demonstrating that bexarotene can be neuroprotective and can reduce intraneuronal accumulations of APP and β-CTF (Fig. 3). Furthermore, these data have begun to identify additional mechanisms of bexarotene in addition to reverse cholesterol transport.

Murine models of AD exhibit a robust inflammatory response and increased levels of pro-inflammatory cytokines which are postulated to contribute to neuronal death^52^,^53^,^54^. TNFα, IL-1β, and IL-6 are pro-inflammatory cytokines secreted by microglia and astrocytes, and have been shown to cause neuronal dysfunction and death^53^,^54^,^55^,^56^. Nuclear receptors act to suppress proinflammatory gene expression through transrepression of NF-κB^5^,^57^, as well as inducing lipid-dependent suppression of inflammation signaling pathways^58^,^59^. Our data indicate that bexarotene treatment suppresses inflammation in astrocytes early in pathology (Fig. 7). Although bexarotene treatment did not decrease microgliosis in the 5XFAD mice, it reduced the overall mRNA levels of proinflammatory cytokines TNFα, IL-1β, and IL-6 (Fig. 7). The reduced presence of TNFα, IL-1β, and IL-6 in the neuron microenvironment may have improved neuron survival and increased resistance to glutamate excitotoxicity^60^. However, we cannot yet clearly distinguish the direct neuronal actions of bexarotene from the glial actions of bexarotene. Parsing apart the mechanism of neuronal actions of bexarotene remains a primary objective of future work with implications on subsequent bexarotene clinical trials^20^.

Overall, we report that bexarotene has neuroprotective effects in an aggressive model of AD which correlated with reduced intraneuronal APP and improved synaptic protein levels. It will be important to further parse apart the mechanisms of neuroprotection since a large variety of pathways have been reported to be altered in response to bexarotene treatment.

## Materials and Methods

### Animals and drug treatment

5XFAD male mice were a gift from Dr. Robert Vassar (Northwestern University) and B6SJL/F1 females were purchased from Jackson Laboratories (Bar Harbor, Maine, USA). Bexarotene (Targretin^TM;^ Valaent Pharmaceuticals, Laval Quebec) was dispersed in water and administered by oral gavage at the dose of 100 mg/kg daily as previously described^8^. Vehicle treated mice received water only. Treatment began at either 3.5 or 7.5 months of age and continued for 15 days. The animals were sacrificed at the end of the treatment period 6 hours after the last dose. Treatment groups were composed of gender balanced littermates (n = 9–14), randomly assigned to treatment. All animal experimentation was approved by the Case Western Reserve University Institutional Animal Care and Use Committee (IACUC). All animal procedures were carried out in accordance with guidelines of IACUC and AAALAC International, which has an Animal Welfare Assurance (A3145–01) on file with the Office of Laboratory Animals.

### Immunohistochemistry

At the end of the treatment period the mice were anesthetized with Avertin and transcardially perfused with 0.1 M phosphate buffered saline (PBS) pH 7.4. Brains were removed, and the right hemisphere was immersed in 4% PFA in 0.1 M phosphate buffer (PB, pH 7.4) for 18 hours at 4 °C, while the left hemisphere was processed for RNA and protein analysis. Brains were cryoprotected in 0.1 M PB (pH 7.4) with 10% sucrose for 24 hours followed by 24 hours in 0.1 M PB with 30% sucrose. Subsequently, the brains were frozen and cut in serial 10 μM parasagittal sections, as described previously^7^. The sections were incubated with antibodies to glial fibrillary acidic protein (GFAP; 1:1000 dilution, DAKO), ionized calcium binding adaptor molecule 1 (Iba-1; 1:1000 dilution, Wako Chemicals), NeuN (1:1000 dilution, Wako Chemicals), 6E10 (1:1000 dilution Biolegend), AB40 (1:1000 dilution, Invitrogen), AB42 (1:1000 dilution, Invitrogen), N-terminal APP antibody (1:1000 dilution, Invitrogen), MOAB-2 (1:1000 dilution, Abcam), APP β-CTF (1:1000 dilution, Sigma), or anti-APLP2 (1:1000 dilution, gift from Dr. Bruce Lamb), followed by incubation with appropriate AlexaFluor 488 or 546 conjugated secondary antibodies (Molecular Probes/Life Technologies, Grand Island, NY, USA).

Images of the cortex and subiculum were obtained from both medial and lateral brain sections. Six images were averaged from the cortex and four images were averaged from the subiculum per slide, totaling twelve images of the cortex and eight of the subiculum per mouse. Immunoreactivity was quantified with ImagePro Premium (Media Cybernetics, Rockville, MD, USA) by a user blinded to the study groups. NeuN positive neurons were manually counted, using Photoshop, in the subiculum and cortical layer V from each animal and normalized to area by an observer blinded to genotype and treatment. Cortical 6E10 immunoreactive neuronal cell bodies were also counted manually the same protocol. Intensity of the intraneuronal 6E10 immunoreactivity was evaluated by imaging the sections at 20X on a confocal microscope and were subsequently quantified using ImageJ by outlining all individual 6E10 immunopositive neurons from 3 separate images of cortical layer V taken from 3 consecutive sections for a total of 60–90 individual neurons per mouse.

### Tissue dissection

The left hemispheres were dissected so that only cortices and hippocampi remained. Hemibrains were homogenized in 800 μl of tissue homogenization buffer (250 mM sucrose, 20 mM Tris pH 7.4, 1 mM EDTA, 1 mM EGTA in diethylpyrocarbonate treated water) containing Protease Inhibitor Cocktail (1:100, Sigma). The homogenates were centrifuged at 5000 g for 10 minutes at 4 °C and supernatants stored at −80 °C and used for western blot analysis.

### Western blotting

Protein concentration of the brain lysates was determined by BCA (Pierce). Equal amounts of protein were run on Bis-Tris 4–12% gels (Life Technologies). The following antibodies were used: anti-actin (Santa Cruz Biotechnology, Dallas, TX, USA); anti-ApoE (Santa Cruz Biotechnology); anti-β-actin (Santa Cruz Biotechnology); anti-ATP-binding cassette transporter A1 (ABCA1; Novus Biologicals, Littleton, CO, USA) and G1 (ABCG1; Novus Biologicals), PSD95, synaptophysin, 6E10 followed by incubation with HRP conjugated secondary antibodies.

### Primary cortical neuronal cultures and Glutamate Excitotoxicity Assay

Primary neurons were cultured as previously described by Malm *et al*.^7^. Cortices of embryonic day 15 5XFAD and C57B6/SJL littermate pups were dissected and freed from meninges. Pups were cultured separately and tailed for genotyping to determine if they were 5XFAD or non-transgenic. After dissociation with 0.025% (w/v) trypsin in Krebs Buffer (0.126 M NaCl, 2.5 mM KCl, 25 mM NaHCO3. 1.2 mM NaH2PO4. 1.2 mM MgCl2. 2.5 mM CaCl2, pH 7.4) for 15 minutes at +37 °C the tissue pieces were treated with 0.008% w/v DNaseI and 0.026% w/v trypsin inhibitor (Sigma, St. Louis, Missouri, USA) and centrifuged at 300 g for 3 minutes. The cell pellet was resuspended in 1 ml of DNaseI/SBT1 (Sigma) in Krebs solution and gently triturated. One ml of additional Krebs buffer was added, the cell suspension centrifuged at 300 g for 3 minutes and the cells were resuspended in Neurobasal medium (Gibco/Life Technologies) supplemented with 0.2 mM L-Glutamine (Gibco), 0.01 mg/mL Gentamycin (Sigma) and B27 Supplement (Gibco), filtered through 100 uM nylon mesh filter, and counted using a haemocytometer. Primary cortical neurons were plated onto poly-D-lysine (50 μg/ml in water) and laminin (5 μg/ml water, Sigma) in 6-well plates at the density of 1 × 10^6^ cells per well. After 7 days *in vitro* the cells were pre-exposed to 5 uM bexarotene or fresh Neurobasal media. After 24 hours, neurons were exposed to 250 uM glutamate in the presence or absence of 5 uM bexarotene for 24 hours. Cell death was measured by MTT assay.

### qRT-PCR

Approximately 125ul RNABee (TelTest Inc, Friendwood, TX, USA) was added to 125 μl of brain homogenate which was then snap frozen on dry ice and stored at −80 °C. After thawing on ice, 200 μl of chloroform (Sigma) was added to the RNA-brain homogenate mixture and was centrifuged for 15 minutes at 13 000 g at 4 °C. The aqueous layer was removed and placed in an RNease filter tube and an equal amount of 70% ethanol was added as directed by the RNease Mini kit (Qiagen). All subsequent kit directions were followed, and mRNA concentration and purity was determined using a NanoDrop 2000 (Thermo Scientific). Equivalent amounts of mRNA were reverse transcribed using a QuantiTect Reverse Transcription kit (Qiagen) according to the manufacturer’s instructions. The cDNA was preamplified for 14 cycles using a TagMan PreAmp Master Mix for select primer sets (Applied Biosystems/Life Technologies). Quantitative PCR was performed with the StepOne Plus Real Time PCR system (Applied Biosystems) for 40 cycles. Analysis of gene expression was performed using the comparative Ct method (∆∆CT) where the threshold cycle for the target genes was normalized to GAPDH and rRNA internal housekeeping gene controls (∆CT). The mRNA expression was presented as fold change and statistical analyses were performed on ∆CT ± SEM for each target gene as described earlier^7^.

### Olfactory Habituation

5XFAD mice (3.5 or 7.5 months) were orally gavaged for 13 days with 100 mg/kg/day bexarotene. Odorants (heptanone, isoamyl acetate, limonene, and ethyl valerate; Sigma Aldrich, St. Louis, MO) were diluted 1 × 10^−3^ in mineral oil and applied to a cotton-applicator stick enclosed in a piece of plastic tubing^32^. Odors were delivered for 4 successive trials (1 block), 20 seconds each, separated by 30 second inter-trial intervals, by inserting the odor stick into a port on the side of the animal’s home cage at day 13 of bexarotene treatment^32^. Testing took place during the light phase of the animals’ (12:12) light:dark cycle. The duration of time spent investigating was defined as snout-oriented sniffing within 1 cm of the odor presentation port, and was recorded across all trials by a single observer blinded to genotype and treatment.

### Remote memory stabilization

As previously reported, 5XFAD mice exhibit normal reconsolidation of contextual fear memory^31^,^61^. Therefore, we chose to examine the well characterized remote memory stabilization protocol established by Kimura and colleagues in this model^31^. Briefly, experiments were performed using two standard conditioning chambers, each of which was housed in an isolation cubicle and equipped with a stainless-steel grid floor connected to a solid-state shock scrambler. Each scrambler was connected to an electronic constant-current shock source that was controlled via an interface connected to a Windows XP computer running FreezeFrame software (Coulbourn Instruments, Allentown, PA). A digital camera was mounted on the side of each chamber, and video signals were sent to the same computer for analysis. During training, mice were placed in the conditioning chamber for 12 min and then received four footshocks (1.0 mA, 2 s). Hippocampus-dependent contextual fear memory formation and the subsequent remote memory stabilization were evaluated by scoring freezing behavior for 5 min when the mice were placed back into the same conditioning chamber 14 days after training.

### Statistics

Significance between groups was determined by a one-way ANOVA with repeated measures, and post-hoc Tukey tests were performed when appropriate. Paired t-tests were used to compare plaque area and number. Data were presented as mean ± SEM and the level of significance was set for p values less than 0.05.

## Additional Information

**How to cite this article:** Mariani, M. M. *et al*. Neuronally-directed effects of RXR activation in a mouse model of Alzheimer’s disease. *Sci. Rep.* **7**, 42270; doi: 10.1038/srep42270 (2017).

**Publisher's note:** Springer Nature remains neutral with regard to jurisdictional claims in published maps and institutional affiliations.

## Supplementary Material

Supplementary Information
