# Supplementary material for: Neuronally-directed effects of RXR activation in a mouse model of Alzheimer’s disease
Source: Sci Rep. 2017 Feb 16;7:42270. doi: 10.1038/srep42270 (PMC5311933; doi:10.1038/srep42270)
Supplement: Supplementary Information [file srep42270-s1.doc]

**SREP-16-21965A**

**Neuronally-directed effects of RXR activation in a mouse model of Alzheimer’s disease**

M. M. Mariani*, T. Malm^+^, R. Lamb, T. R. Jay, L. Neilson, B. Casali, L. Medarametla & G.E. Landreth

**Supplemental Figure 1 Bexarotene does not significantly alter endosomal trafficking markers or autophagy initiation markers.** A) 5XFAD mice were treated for 15 days with vehicle or Bexarotene. Combined cortical and hippocampal homogenates were probed for Rab7, Rab5, p62/SQSTM1, and LC3-II levels by Western blot. One-way ANOVA *p <0.05 (n = 4-5)


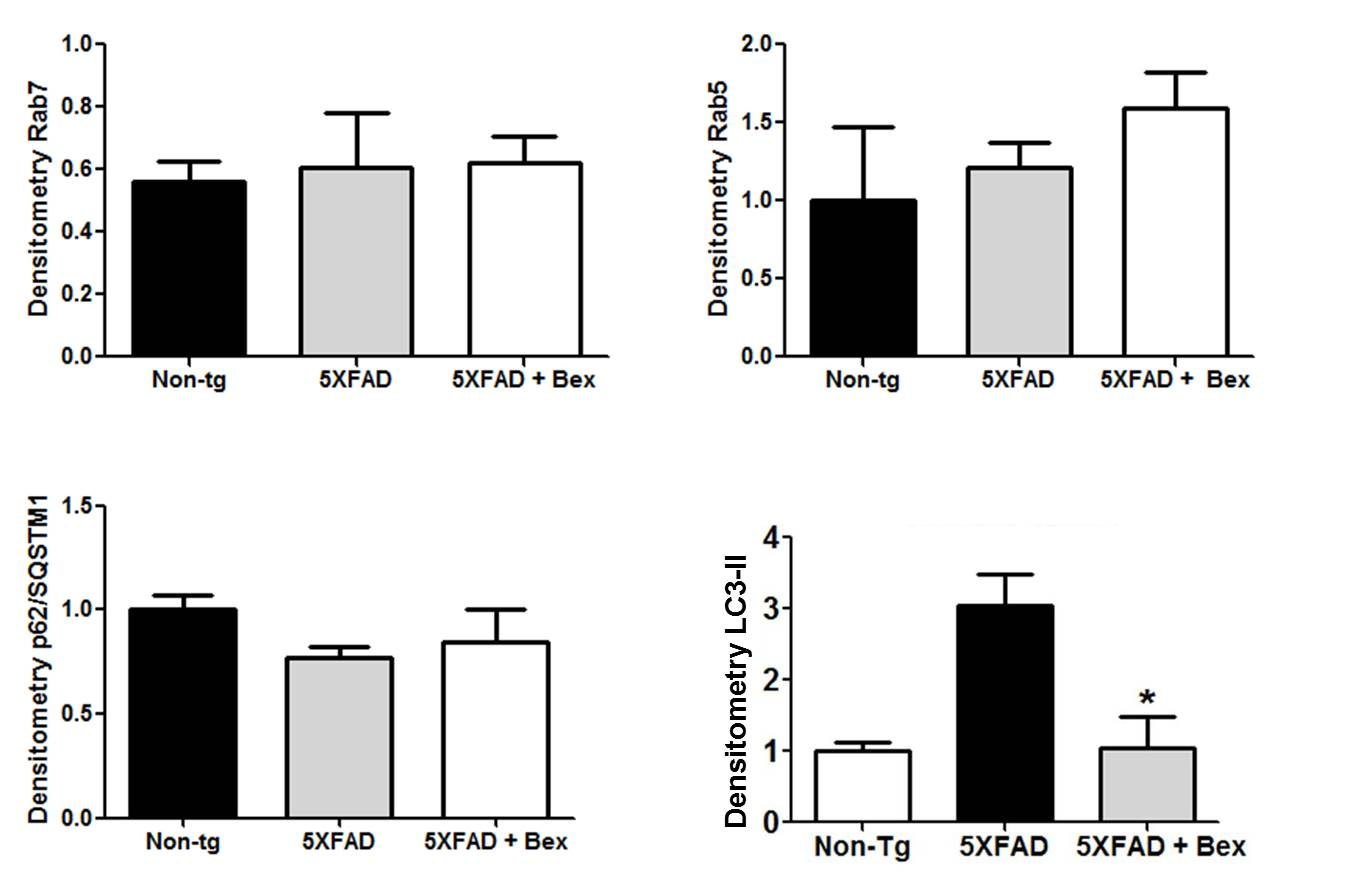


**Supplemental Figure 2 Bexarotene reduces plaque area and number in 5XFAD mice.** A) Quantification of plaque levels in 4 month and 8 month 5XFAD mice treated for 15 days with bexarotene or vehicle and stained with 6E10. One-way ANOVA *p <0.05 B) 4 month 5XFAD hippocampal and cortical homogenates were analyzed for the expression of APOE and lipidation of APOE by Western blot and native blot, respectively. Analysis and quantification was performed with Image J. Student’s T test, *p <0.05


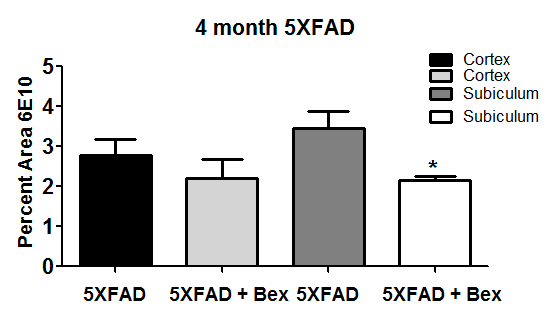


A)


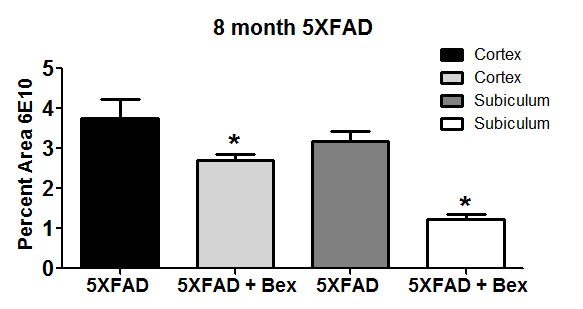


B)


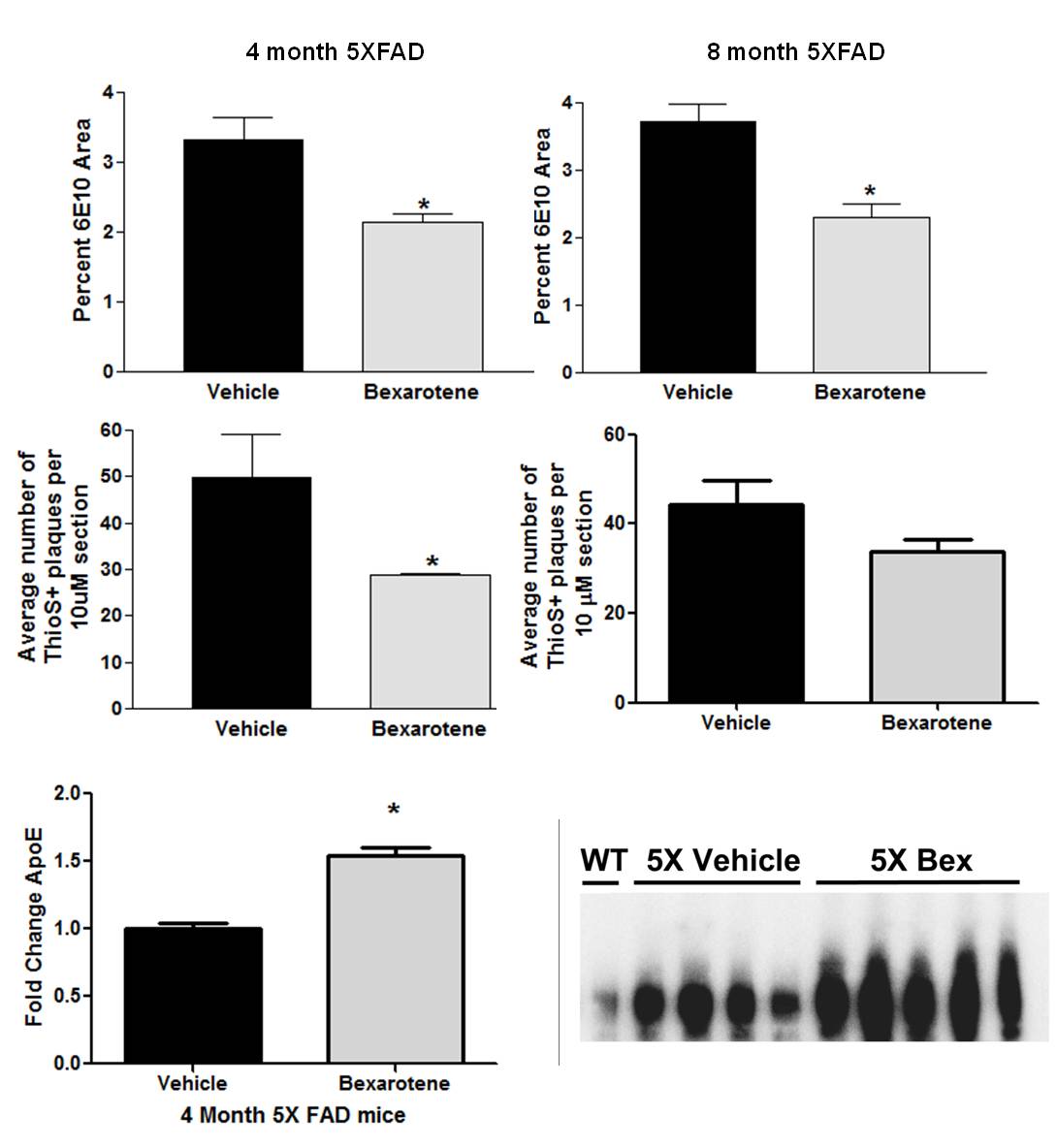

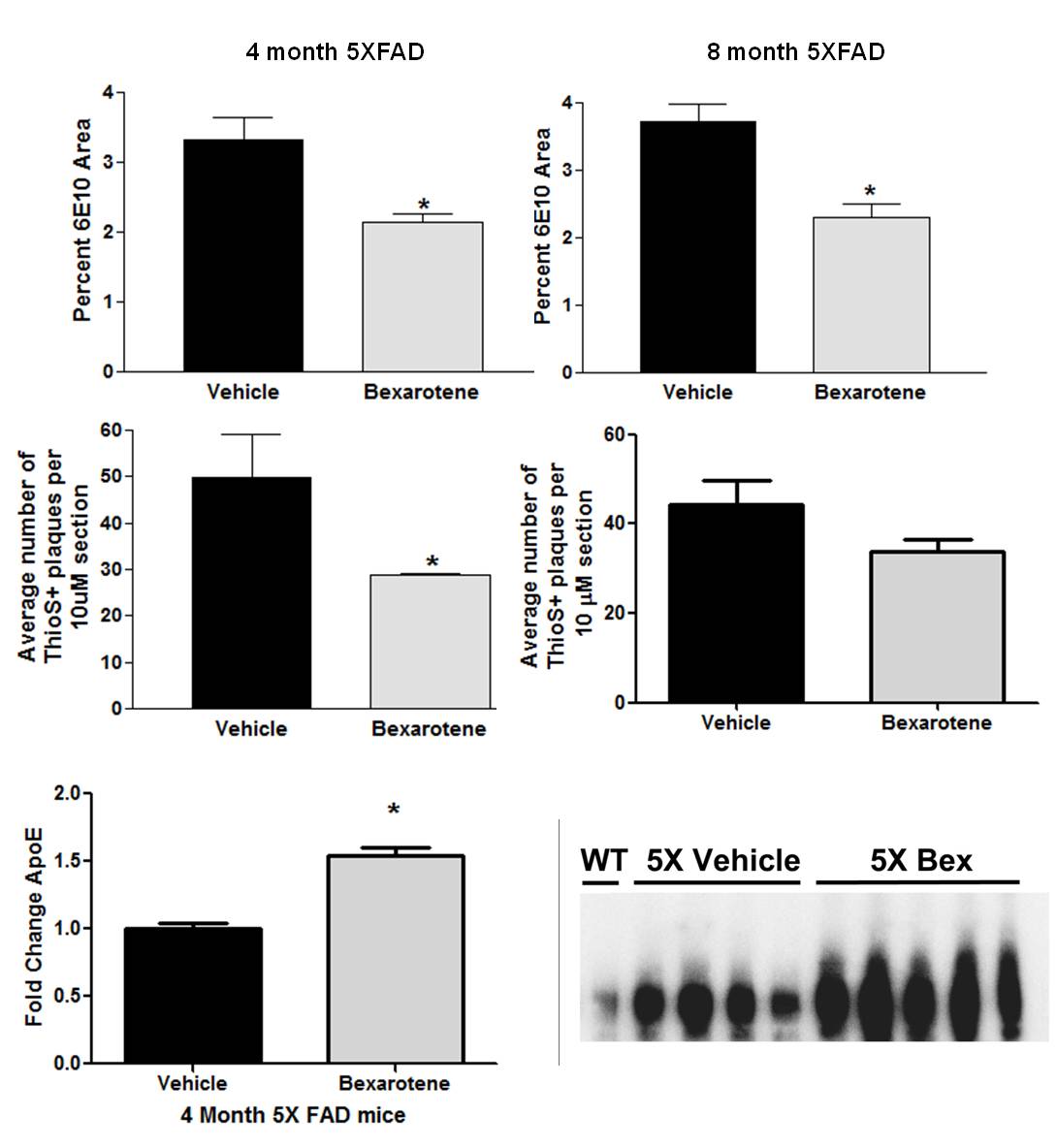


**Supplemental Figure 3 Bexarotene reduces Aβ40 in late AD pathology.** 5XFAD brain homogenates from A) 4 month or B) 8 month old mice were DEA (soluble fraction) and subsequently FA (insoluble fraction) extracted. Combined cortical and hippocampal Aβ levels were quantified using ELISA for Aβ42 or Aβ40 and normalized to total protein. One-way ANOVA, *p <0.05 (n = 7-9 for 4 month and 8 month)

**
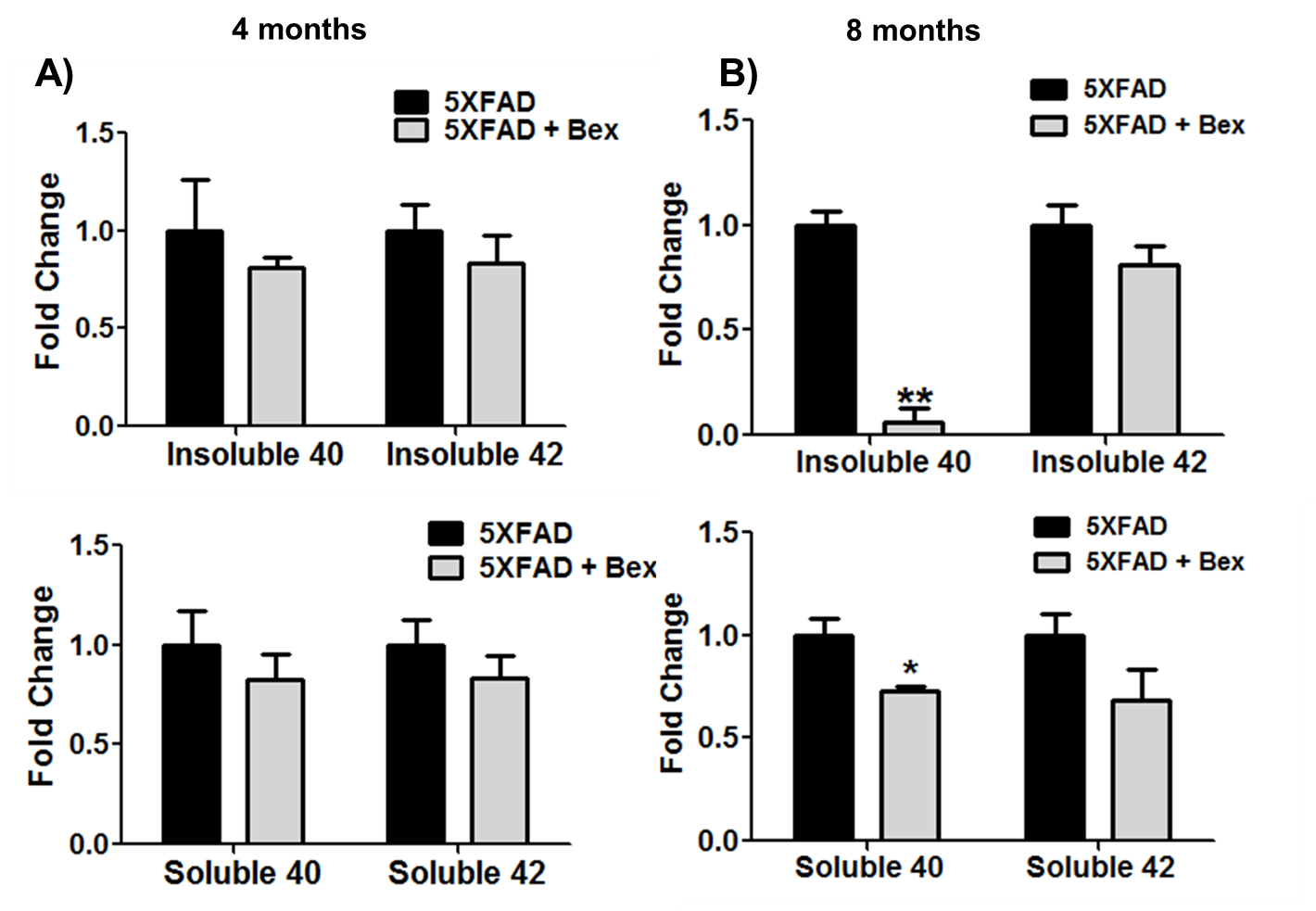
**
